# Selection of reference genes for gene expression studies in ultraviolet B-irradiated human skin fibroblasts using quantitative real-time PCR

**DOI:** 10.1186/1471-2199-12-8

**Published:** 2011-02-17

**Authors:** Li Li, Yan Yan, Haoxiang Xu, Tao Qu, Baoxi Wang

**Affiliations:** 1 Department of Dermatology, Peking Union Medical College Hospital, Chinese Academy of Medical Sciences & Peking Union Medical College, Beijing, PR China; 2 Chinese Academy of Medical Sciences and Peking Union Medical College Institute of Dermatology, Nanjing, PR China

## Abstract

**Background:**

Reference genes are frequently used to normalise mRNA levels between different samples. The expression level of these genes, however, may vary between tissues or cells and may change under certain circumstances. Cytoskeleton genes have served as multifunctional tools for experimental studies as reference genes. Our previous studies have demonstrated that the expression of vimentin, one cytoskeletal protein, was increased in ultraviolet B (UVB)-irradiated fibroblasts. Thus, we examined the expression of other cytoskeleton protein genes, *ACTB *(*actin, beta*), *TUBA1A *(*tubulin, alpha 1a*), and *TUBB1 *(*tubulin, beta 1*), in human dermal fibroblasts irradiated by UVB to determine which of these candidates were the most appropriate reference genes.

**Results:**

Quantitative real-time PCR followed by analysis with the NormFinder and geNorm software programmes was performed. The initial screening of the expression patterns demonstrated that the expression of *VIM *was suppressed after UVB irradiation at doses ≥25 mJ/cm^2 ^and that the expression of *TUBA1A *was significantly reduced by UVB doses ≥75 mJ/cm^2 ^in cultured human dermal fibroblasts. The analysis of the experimental data revealed *ACTB *to be the most stably expressed gene, followed by *GAPDH *(*aglyceraldehyde-3-phosphate dehydrogenase*), under these experimental conditions. By contrast, *VIM *was found to be the least stable gene. The combination of *ACTB *and *TUBB1 *was revealed to be the gene pair that introduced the least systematic error into the data normalisation.

**Conclusion:**

The data herein provide evidence that *ACTB *and *TUBB1 *are suitable reference genes in human skin fibroblasts irradiated by UVB, whereas *VIM *and *TUBA1A *are not and should therefore be excluded as reference genes in any gene expression studies involving UVB-irradiated human skin fibroblasts.

## Background

Ultraviolet B (UVB) radiation is the most active, albeit minor, constituent of solar light. UVB has both direct and indirect adverse biological effects that may result in photo-aging and photo-carcinogenesis. The DNA damage caused by UVB irradiation is considered to be responsible for basal cell carcinoma and squamous cell carcinoma [1,2]. UVB is also suspected of lowering the immune defence system of the skin [3]. Given these effects, the gene expression of dermal fibroblasts after UVB irradiation has become a significant area of study, with a total of 384 manuscripts found in PubMed using the keyword 'UVB' just within the last year.

Quantitative real-time PCR (qPCR) is the most powerful method used to quantify gene expression. Similar to other expression study methods, the sample data are usually required to be normalised against either another data set or particular references to correct for any differences in the amount of starting material. At present, the most common normalisation method involves the use of a single internal control reference gene, often selected from a set of genes referred to as 'housekeeping' genes that are constitutively expressed in certain tissues and/or under certain circumstances. There is strong evidence in the literature, however, to suggest that the expression of some of these reference genes may be constant under certain conditions but may also fluctuate significantly under other conditions [4,5]. Commonly accepted reference genes, such as *ACTB *(*actin, beta*) and *GAPDH *(*aglyceraldehyde-3-phosphate dehydrogenase*), have been shown to be affected by particular *in vitro *experimental conditions and some clinical conditions, such as asthma [6], thus indicating that they may not always be suitable candidates for normalisation [7]. The normalisation of data using a non-validated reference gene could lead to inaccurate results and therefore erroneous conclusions. Previous studies have reinforced the need to validate reference genes prior to their use in a study. Cytoskeletal protein genes, including *ACTB*, *TUBA1A *(*tubulin, alpha 1a*), and *TUBB1 *(*tubulin, beta 1*), have been used as reference genes for many experimental conditions.

The cytoskeleton is the cellular 'scaffolding' or 'skeleton' that is contained within the cytoplasm. Its concept and term (*cytosquelette*, in French) were first introduced by the French embryologist Paul Wintrebert in 1931 [8]. The cytoskeleton is composed of three types of protein filaments: actin filaments, intermediate filaments and microtubules [9]. The cytoskeleton was once thought to be unique to eukaryotes, but recent research has identified a prokaryotic cytoskeleton [10]. The cytoskeleton is a dynamic structure that maintains cell shape, protects the cell, enables cellular movement, and plays important roles in both intracellular transport and cell division [11,12]. Not only is it an indispensable protein complex for all cells, including eukaryotes and prokaryotes, but it also serves as a multifunctional tool because many of its proteins can be used as reference genes for a variety of experimental studies.

Our previous studies have demonstrated that fibroblasts increased the expression of vimentin, a cytoskeleton protein, after irradiation by UVB [13]. Whether the expression levels of other cytoskeletal genes, such as *ACTB*, *TUBA1A*, and *TUBB1*, are altered after UVB irradiation and consequently whether any of these genes are suitable reference genes remain unknown. In this study, primary human dermal fibroblasts were treated with different doses of UVB radiation, and the irradiated cells were analysed at different time points. The gene expression of four cytoskeletal genes (Table 1) after irradiation was compared with another common reference gene, *GAPDH*. These five genes were also analysed with respect to their suitability as reference genes for expression studies in healthy and irradiated fibroblasts. To define the most stable reference genes for these study conditions, the expression changes between and within these two groups were investigated and analysed using two software products available free online.

**Table 1:** Characteristics of the five reference gene candidates analysed in this study.

Gene symbol	Gene name	Accession No.	Function	Gene aliases	Locus
*GAPDH*	aglyceraldehyde-3-phosphate dehydrogenase	NM_002046.3	Glycolytic enzyme	G3PD;GAPD	12p13
*ACTB*	actin, beta	NM_001101.3	The contractile apparatus and nonmuscle cytoskeletal actins	PS1TP5BP1	7p15-p12
*TUBA1A*	tubulin, alpha 1a	NM_006009.2	The components of microtubules	LIS3; TUBA3; FLJ25113; B-ALPHA-1	12q12-q14.3
*TUBB1*	tubulin, beta 1	NM_030773.2	the components of microtubules	dJ543J19.4	20q13.32
*VIM*	vimentin	NM_003380.2	maintaining cell shape, integrity of the cytoplasm	FLJ36605	10p13

## Results

### Assessment of pre-analytical and analytical variables

**Figure 1:**
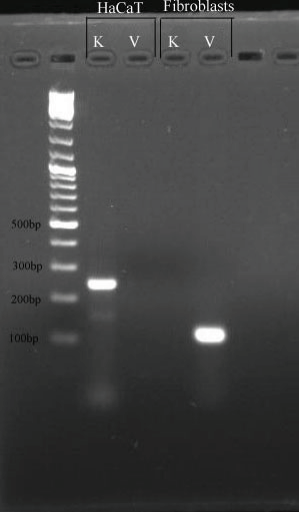
**Detection of *VIM *and *keratin 10 *by standard PCR in primary fibroblasts and the HaCaT cell line**. V: the expression of *VIM*, K: the expression of *keratin 10*.

**Figure 2:**
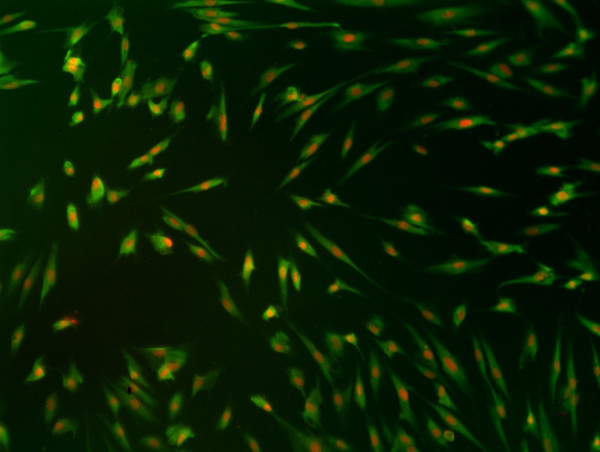
**Vimentin expression in cultured human dermal fibroblasts**. Vimentin staining (green) was positive by immunohistochemical analysis, showing its localisation to the nucleus. The cytoplasm was stained orange. Magnification = ×20

The determination of the primary cell type was the basis of these experiments. Fibroblasts express vimentin, whereas keratinocytes express keratin. Thus, the cells isolated from the foreskin samples were examined to determine if vimentin and/or keratin were expressed at both the mRNA transcript and protein levels. In fibroblasts, PCR results in a bright, specific band for the vimentin primers but no specific products for the keratin10 primers by 2% agarose gel electrophoresis analysis. The opposite pattern was seen in the HaCaT keratinocyte cell line (Figure 1). Fibre bundles in the cytoplasm along the cell axis stained green, while the nucleus stained orange by fluorescent immunohistochemical analysis in fibroblasts(Figure 2). These same fibroblasts were negative for the expression of the keratin protein (data not shown). Taken together, these results indicated that highly pure human dermal fibroblasts were successfully obtained from the foreskin samples.

In RT-qPCR experiments, there are many pre-analytical variables, including the collection and storage of samples and the RNA isolation method, that determine the quality of the RNA that is used for the subsequent quantitative expression analyses. Therefore, the RNA samples isolated from the 12 matched normal and irradiated fibroblasts were characterised with respect to their concentration, purity, and integrity. Only high quality RNA samples were included in this study to avoid erroneous conclusions. The mean A260/280 ratio of the RNA samples was 2.01 ± 0.037 (range: 1.93 - 2.08), reflecting pure and protein-free RNA. The RNA integrity as an essential quality criterion was characterised using the so-called RNA integrity number (RIN), measured by the Agilent 2100 Bioanalyzer. The mean RIN value (± SD) of the RNA samples was 9.3 ± 0.57 (range: 8.0 - 10.0).

Pooled cDNA samples were used as a precision control for each gene-specific PCR run. These controls were adjusted to calculate the PCR efficiency as described in the methods. The mean PCR efficiencies varied from 0.967 to 1.06 (Table 1). The between-run analytical performances of the RT-PCR measurements were determined using these controls, and the between-run variations (n = 5) ranged from 0.17% to 0.38%. The standard deviation (SD) for the triplicate reactions ranged from 0.023 to 0.751 cycles (mean = 0.31 cycles).

### UVB suppressed the expression of *TUBA1A *and *VIM *in cultured human dermal fibroblasts

**Figure 3:**
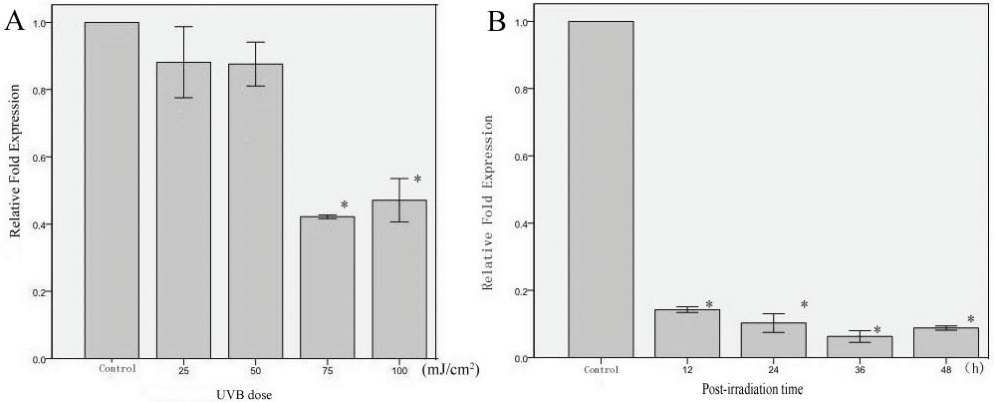
**The relative fold expression of *TUBA1A *in fibroblasts irradiated by UVB at the indicated time points**. (Figure 3-A) *TUBA1A *expression in fibroblasts 24 h after treatment with different does of UVB, including 0, 25, 50, 75, and 100 mJ/cm^2^. (Figure 3-B) The time course of *TUBA1A *expression after treatment with 125 mJ/cm^2 ^UVB. The error bars represent the standard deviation. The experiments were performed in triplicate. * *p < 0.05 *compared with the control group.

**Figure 4:**
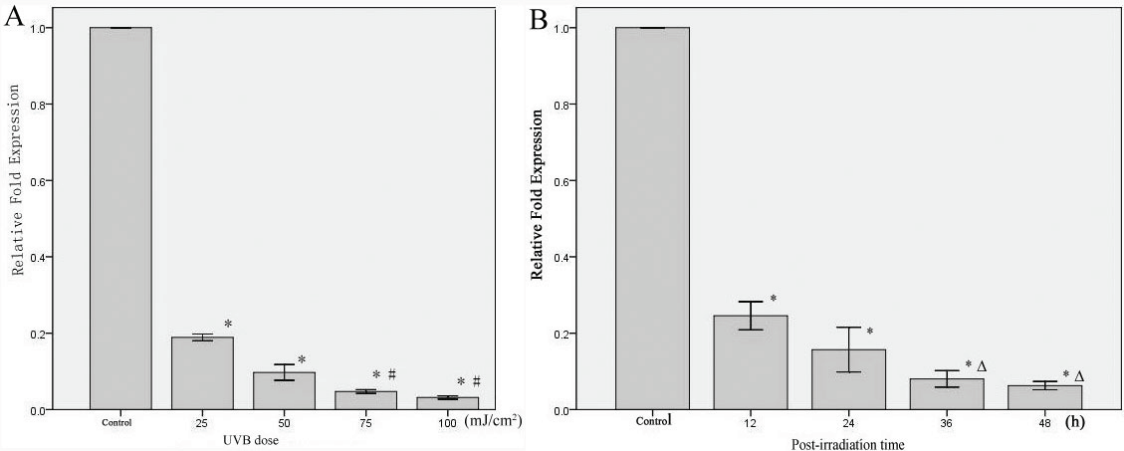
**The relative fold expression of *VIM *in fibroblasts irradiated by UVB at the indicated time points**. (Figure 4-A) *VIM *expression in fibroblasts 24 h after treatment with different does of UVB, including 0, 25, 50, 75, and 100 mJ/cm^2^. (Figure 4-B) The time course of *VIM *expression after treatment with 125 mJ/cm^2 ^UVB. The error bars represent the standard deviation. The experiments were performed in triplicate. * *p < 0.05 *compared with the control groups; *# p < 0.05 *compared with the group irradiated with a dose of 50 mJ/cm^2^; Δ *p < 0.05 *compared with the group 24 h after exposure.

To determine whether UVB affects the expression of cytoskeletal genes in cultured human fibroblasts, the fibroblasts were exposed to various doses of UVB (25, 50, 75, and 100 mJ/cm^2^). After UVB irradiation, the fibroblasts were cultured for 24 h, and then total RNA was extracted from the cells. qPCR was performed to determine the changes in the expression of these cytoskeletal genes. As common reference genes, the expression levels of *ACTB*, *GAPDH *and *TUBB1 *were not significantly different between the normal and irradiated fibroblasts (data not shown). Doses of 25 and 50 mJ/cm^2 ^did not alter the expression levels of *TUBA1A *(Figure 3-A), but they were significantly reduced at doses ≥75 mJ/cm^2 ^(*P < 0.05*). The expression of *VIM *began to be significantly reduced after exposure to a UVB dose of 25 mJ/cm^2 ^(*P < 0.05*; Figure 4-A). The decreased expression of *VIM *became even more pronounced at doses of 75 and 100 mJ/cm^2 ^when compared with 25 and 50 mJ/cm^2 ^(*P < 0.05*). The mRNA expression levels of *TUBA1A *and *VIM *were reduced by varied UVB intensities.

To determine whether the time after exposure to UVB affects the expression of cytoskeletal protein genes in cultured human fibroblasts, total RNA was extracted at different time points (12, 24, 36, and 48 h) after exposure to a 125 mJ/cm^2 ^UVB dose. This dose did not have a significant effect on the expression levels of *GAPDH*, *ACTB *and *TUBB1 *at the time points up to 48 h post-irradiation (data not shown). The expression of *TUBA1A *didn't show statistically significant difference at the 12, 24, 36, 48 h though it was decreased by 125 mJ/cm^2 ^of UVB (Figure 3-B). T. The *VIM *mRNA level decreased after exposure to a UVB dose of 125 mJ/cm^2 ^(*P < 0.05*), and this decrease appeared to be time dependent, with greater decreases seen after 36 and 48 h compared with those seen at 12 and 24 h post-exposure (*P < 0.05*; Figure 4-B). Thus, neither *TUBA1A *nor *VIM *is a suitable reference gene for research involving UVB-irradiated human fibroblasts.

### Expression levels and ranges of the reference gene candidates

**Figure 5:**
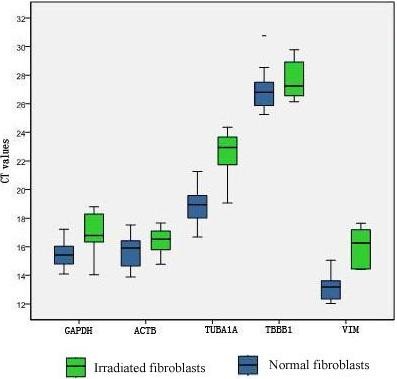
**Expression levels of candidate reference genes in normal (blue boxes) and irradiated (green boxes) human skin fibroblasts**. The data are presented as real-time PCR cycle threshold (Ct) values. The horizontal line represents the median Ct value, and the boxes and whiskers represent the inter-quartile and min-max ranges, respectively.

Although the expression levels of *TUBA1A *and *VIM *were significantly reduced in fibroblasts after exposure to UVB, they were still evaluated as potential reference genes along with *ACTB*, *GAPDH *and *TUBB1*. The expression levels of these five candidates (Table 2), shown in terms of Ct-values, are given as box-and-whisker plots in Figure 5. The boxes represent the median Ct-values and the inter-quartile ranges, while the whiskers indicate the min-max ranges. Genes with higher expression levels show lower Ct-values under the specific PCR conditions, and genes with lower expression levels have inversely higher cycle numbers. The five candidate reference genes showed a wide range of expression levels with Ct values falling between 12 and 34 cycles. The median Ct values were in the range usually covered by reference genes, varying from 16.48 (*GAPDH*) to 29.75 (*TUBB1*). The genes could be divided into two groups: a group of genes with a median Ct value below 20 (*GAPDH and ACTB*, *VIM*) or above 20 (*TUBA1A*, *TUBB1*) (Figure 5). There was a trend of higher median Ct values of all reference genes in irradiated fibroblasts compared to normal fibroblasts. However, the difference was not big enough to conclude that the *ACTB*, *GAPDH*, and *TUBB1 *expression levels differed between normal and irradiated fibroblasts. *TUBA1A *and *VIM *had significantly different expression levels in normal and irradiated fibroblasts (*P < 0.01*). Individual candidate reference genes also showed different expression ranges across all studied samples. *TUBB1 *and ACTB showed the smallest Ct value variability in normal (min-max range: 1.17) and irradiated (min-max range: 3.4) fibroblasts, respectively, while *TUBA1A *(min-max range: 3) and *TUBA1A *(min-max range: 7.07) showed the largest variance in normal and irradiated fibroblasts.

**Table 2:** Sequences and related information of the primers

Gene symbol	**Primer sequence**^**a**^	Amplicon length (bp)	Annealing temperature (°C)	**PCR efficiency**^**b**^**/E**	R
*GAPDH*	F: ACAGTCAGCCGCATCTTCTT R: GTTAAAAGCAGCCCTGGTGA	127	57	1.001	0.999
*ACTB*	F: GGCGGCAACACCATGTACCCT R: AGGGGCCGGACTCGTCATACT	205	62	1.06	0.95
*TUBA1A*	F: GCAACAACCTCTCCTCTTCG R: GAATCATCTCCTCCCCCAAT	200	57	1.043	0.991
*TUBB1*	F: CCTTGCAGCTGGAGAGAATC R: CTGTCGGGTTGAAAGAGAGC	145	59	0.967	1.00
*VIM*	F: GACAGGATGTTGACAATGCG R: GTTCCTGAATCTGAGCCTGC	135	59	1.03	0.998
*K10*	F: GCCAACATCCTGCTTCAGAT R: TCACATCACCAGTGGACACA	256	58		

^a ^All primer sequences are given in the 5'→3' direction.

^b ^PCR efficiencies were determined by PCR DataMiner and confirmed by a dilution series experiment.

### Expression stability of reference gene candidates

**Figure 6:**
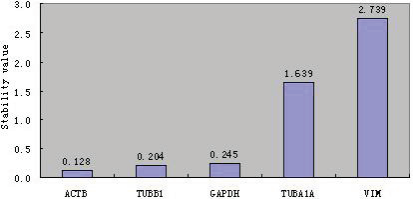
**The average value of expression stability of each gene determined using the NormFinder software**.

The NormFinder algorithm was used to rank the five irradiated fibroblast reference gene candidates according to their expression stability (Figure 6). As revealed by the analysis, *ACTB*, characterised by the stability value of 0.128, had the most stable expression levels and thus was selected by the algorithm as the best choice for a single reference gene for expression studies in normal and irradiated fibroblasts. *TUBB1*and *GAPDH *were ranked as the second- and third-best reference genes, respectively. Two commonly used reference genes, *TUBA1A *and *VIM*, were ranked last, suggesting that they should not be considered as reliable reference genes for expression assay studies involving fibroblasts. The programme additionally identified *ACTB *and *TUBB1 *as the best combination of two reference genes.

NormFinder can also be used to calculate the intragroup variation. The variation of the five candidate genes in normal and matched irradiated fibroblast pairs are presented in Table 3. The variation of *ACTB *in the normal and matched irradiated fibroblasts was the lowest, whereas *TUBA1A *and *VIM *had the largest variance. The expression of *VIM *began to be significantly reduced after exposure to a UVB dose of 25 mJ/cm^2^, with an even more pronounced reduction at 75 and 100 mJ/cm^2^, which may explain the variance of *VIM *expression seen in the irradiated fibroblasts.

**Table 3:** The intragroup variation as determined by the Norm Finder software for each gene.

	Intragroup variation	
**Gene name**	**Normal fibroblasts**	**Irradiated fibroblasts**
*GAPDH*	0.026	0.511
*ACTB*	0.012	0.036
*TUBA1A*	0.05	5.284
*TUBB1*	0.02	0.046
*VIM*	0.038	7.535

**Figure 7:**
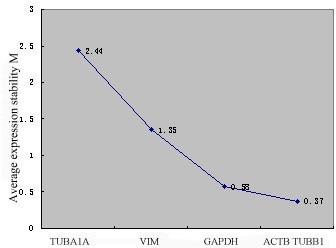
**The stepwise exclusion of least stable genes by calculating the average M value**. The average value of the expression stability M for each gene was determined using geNorm software. Genes on the x-axis are ordered from the left to the right according to their expression stability from the least to the most stable.

The expression level stability of the candidate genes in normal and irradiated fibroblasts was also investigated using the geNorm programme. The curve presented in Figure 7 represents the stepwise exclusion of the least stable candidate reference gene. This algorithm confirmed the results obtained by NormFinder, showing that *TUBA1A *and *VIM *were the least stable reference genes, while the combination of *ACTB *and *TUBB1 *was the most stable gene pair (M = 0.37).

**Figure 8:**
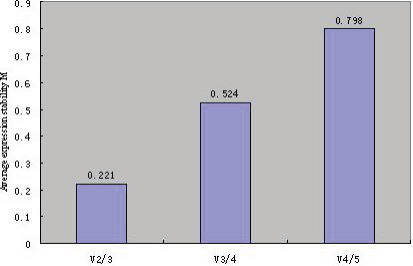
**Evaluation of the optimum number of reference genes according to the geNorm software**. The magnitude of the change in the normalisation factor after the inclusion of an additional reference gene reflects the improvement that is obtained. Vn/n + 1 represents a comparison of the different models, which are those with n and n+1 reference genes.

The geNorm analysis also allows for evaluation of the optimal number of reference genes required for a reliable and accurate normalisation of expression data. It is suggested that the Vn/Vn+1 cut-off value of 0.15 should be considered as a limit beneath which the involvement of additional reference genes would not be required [14]. However, based on our experimental data, the variation Vn/Vn+1 did not reach this cut-off; the observed variability ranged from 0.221 for the addition of a third reference gene (n = 2) to 0.798 for the addition of five reference genes (n = 4) (Figure 8). With the increase in the number of genes, the variation Vn/Vn + 1 did not decrease. This may be related to the changes in *TUBA1A *and *VIM*.

## Discussion

The fluorescence-based quantitative real-time PCR (qPCR) has the capacity to detect and measure minute amounts of nucleic acids in a wide range of samples from numerous sources, enabling it to be the technology par excellence for multiple applications in molecular diagnostics, life sciences, agriculture, and medicine [4,5]. The remarkable lack of consensus on the best qPCR methods for different experiments, however, has the adverse consequence of perpetuating a string of serious shortcomings that encumber its status as an independent yardstick. In 2008, a set of "MIQE" guidelines were proposed to establish a minimum standard for the required information that should be included in publications utilising qPCR experiments [1]. In the experiment described herein, we adhered to the MIQE guidelines to the best of our abilities. The RNA extracted from the primary fibroblasts was validated by immunofluorescence, with its quantity, quality and integrity subsequently determined. All RNA meeting the minimum experimental standards was stored at -80°C and was utilised within two month. Primer specificity was confirmed by melting curve analyses. PCR efficiency plots were calculated using a standard curve with the coefficient of correlation (r) between 0.95 and 1.0, and the PCR efficiency of all primers used in this study varied from 0.967 to 1.06. To ensure consistency between reactions, the same quantity of RNA was used, and both RT and qPCR were performed in triplicate. All of these steps ensured the reliability of this experiment and the comparability of the statistical data that was generated.

Another important factor that may affect qPCR results is the selection of the reference genes. *ACTB*, GAPDH, 18S and 28S rRNA are the most commonly used reference genes, but a number of studies have provided solid evidence that their transcription levels vary significantly among different individuals, cell types, developmental stages, and/or experimental conditions [15,16]. Herein, our aim was to identify genes with the lowest expression variability and validate their use for qPCR data normalisation in expression studies on irradiated fibroblasts, employing two different techniques. To the best of our knowledge, this study is the first to analyse the expression stability of reference gene candidates in fibroblasts exposed to UVB radiation. Our previous studies demonstrated that the expression of vimentin, a cytoskeletal protein, was increased in fibroblasts after UVB irradiation. Thus, this study focused on cytoskeletal proteins commonly used as references, which were *ACTB*, *TUBA1A*, *TUBB1*, and *VIM*, *in addition to *another common reference gene, *GAPDH*.

The two approaches used to analyse the expression stability of the selected reference genes in our study were geNorm and NormFinder. The geNorm application measures gene expression stability (M), which is the mean pairwise variation between an individual gene and all other tested reference genes. During this analysis, the least stable genes (with the highest M values) were sequentially eliminated until only the two most stable genes remained. In addition, the geNorm programme can determine the optimal number of genes required for accurate normalisation by calculating pair-wise variations between consecutively determined normalisation factors of n and n+1 genes [V n/(n+1)]. In the original publication describing geNorm [16], a threshold of 0.15 was established for the pairwise variation, below which the inclusion of additional reference genes is considered to be unnecessary. NormFinder, which is rooted in a mathematical model of gene expression, uses a solid statistical framework to estimate not only the overall expression variation of the normalisation gene candidates, but also the variation between subgroups within a sample set (*e.g.*, normal and cancer samples).

In this study, *ACTB *had the most stable expression, making it the most suitable reference gene for qPCR data normalisation in normal and irradiated fibroblasts. Surprisingly, *GAPDH*, which is one of the most commonly used endogenous controls, was the third most stable gene in this study. There has been an increasing number of reports in the literature documenting the variability of the levels of *GAPDH *mRNA under a variety of physiological and pathological conditions [17,18]. The use of *GAPDH *as a reference gene in the majority of tumour types, including melanoma, has recently been challenged [19]. *TUBA1A *is also commonly used as an endogenous control. In this study, however, the expression of *TUBA1A *decreased after UVB exposure using doses greater than 75 mJ/cm^2^, making it unsuitable as a reference gene for research involving irradiated fibroblasts. Among the examined reference gene candidates, TUBA1A lies in the middle with respect to its suitability as a reference gene. A separate study by Spanier KI determined that *TUBA1A *was a particularly ill-suited reference gene in the Daphnia Pulex genome sequence [20].

In other studies involving qPCR, there was a trend towards the use of more than one reference gene. As described above, the geNorm programme can determine the optimal number of reference genes required for the reliable and accurate normalisation of expression data, with the Vn/Vn+1 cut-off value, which indicates the threshold at which additional reference genes are considered unnecessary, being set at 0.15 [14]. The V2/V3 value of 0.221 obtained in this study was close to the cut-off value of 0.15. The Vn/n+1 value presented an unusual trend in our study, with V2/V3 giving the lowest value. This observation was likely due to the changes seen in the expression levels of *TUBA1A *and *VIM*.

Although our previous studies have shown that the protein expression levels of vimentin were increased in fibroblasts, our results herein showed that the *VIM *mRNA expression level was reduced by UVB irradiation. These two observations appear to contradict each other, and the underlying mechanism for these differences is unclear. Many studies have shown that vimentin cleavage has an impact on the integrity and the dynamics of the intracellular structures that are targets of destruction during apoptosis [21]. Based on that data, we speculate that the over-accumulation of the vimentin protein is one of the factors that contribute to the resistance of skin fibroblasts against environmental stresses. In addition, although *TUBA1A *expression was significantly reduced by exposure to UVB at doses greater than 75 mJ/cm^2^, there was no apparent change in the levels of the *TUBA1A *protein. Unfortunately, these results do not provide any insight as to whether the changes in the *VIM *and *TUBA1A *expression levels correlate with ultraviolet radiation-induced skin damage. Cytoskeleton-deficient cells could be used to detect interactions of the cytoskeleton with the corresponding proteins and the signal transduction pathway involved in the response to UVB irradiation.

## Conclusion

Based on our results, the expression of the *TUBA1A *and *VIM *transcripts decreased in fibroblasts after UVB exposure, and thus, they were not suitable for use as reference genes. Instead, we recommend that *ACTB *be used as a single reference gene for qPCR data normalisation in gene expression studies in normal and irradiated fibroblasts. The addition of *TUBB1 *to *ACTB *for use as a pair of reference genes is recommended for more accurate normalisation. Finally, we emphasise that the utility of the selected reference gene(s) must be confirmed by each research group for each particular experimental setup.

## Methods

### Primary fibroblast culture

Normal human dermal fibroblasts were obtained from the foreskin of healthy donors. The donor group comprised 24 individuals between the ages of 18 and 30 (mean age = 23). All donors provided informed consent prior to their participation in the study. The study protocol was reviewed and approved by the ethics committee of Peking Union Medical College Hospital.

To obtain the fibroblasts, the foreskin was first incubated overnight in 0.25% trypsin. After separation from the epidermis using sterile tweezers, the dermis was cut into pieces to disperse the cells and obtain a single-cell suspension. Cell suspensions from two of the healthy donors were then mixed. The resulting fibroblasts were cultured in Dulbecco's Modified Eagle's Medium (DMEM; Gibco, USA) supplemented with 10% (v/v) heat-inactivated foetal bovine serum (FBS; Bovogen, Australia), 100 U/ml penicillin and 100 mg/ml streptomycin (Invitrogen, USA) in a CO_2 _incubator (5% CO_2_) at 37°C. The experiments were performed on fibroblasts at 3 to 6 passages. For the irradiation experiments, the cells were seeded in 6 cm Petri dishes and grown to 80% confluence.

### Identification of primary fibroblasts and associated transcript and protein levels

Total RNA was extracted from the fibroblasts and reverse-transcribed into cDNA. Primers for *VIM *and *keratin 10 *(*K10*) were designed and synthesised. A detailed description of the primer design process is given below (see '**Primer design**'). Standard PCR was then performed. Briefly, the samples were amplified in 25 μl aliquots containing the cDNA (2 μl), 2× EasyTaq PCR Supermix (12.5 μl; TransGen Biotech, China) and 200 pmol each of the forward and reverse primers. The polymerase was activated at 94°C for 5 min, followed by 45 amplification cycles consisting of a denaturation step at 94°C for 15 s, annealing at a primer-specific temperature (55°C for VIM; 57°C for K10) for 20 s with a ramping rate of 2°C/s, and an extension step at 72°C for 20 s. The specificity analysis of the PCR products was performed immediately after PCR using 2% agarose gel electrophoresis, with the resulting image scanned by the BINTA 2020D gel imaging system (FluorChem SP, USA). RNA extracted from HaCaT cells, which were cultured in DMEM, was used as a positive control for keratin.

The fibroblasts were incubated on coverslips in 24-well plates for at least 48 h. The cells were then fixed in 4% paraformaldehyde at 4°C for 20 min. The coverslips were washed three times in phosphate-buffered saline (PBS) and air dried. For intracellular staining, the cells were permeabilised in 0.15% Triton X-100-5% BSA-PBS at room temperature for 20 min, followed by two extensive washes with PBS. The cells were then incubated in 3% H_2_O_2 _for 10 min to eliminate endogenous peroxidase and again washed in PBS three times. The cells were incubated at 4°C for 2 h with the primary mouse monoclonal anti-vimentin 9 (1:100; Santa Cruz Biotechnology) and anti-keratin (1:100, Santa Cruz Biotechnology) antibodies and then washed twice in PBS-T (0.1% Tween-20) for 10 min. The cells were further incubated with a FITC-labelled sheep anti-mouse IgG secondary antibody (1:100; Vector, USA) and 10 μl propidium iodide at room temperature for 2 h. The stained cells were then washed with PBS-T and visualised using an inverted microscope (Nikon TE300). When the fibroblasts were identified, all the experiment following were preformed three times.

### UVB irradiation

A Waldmann UV 208T (HerbertWaldmann GmbH & Co., Villingen-Schwenningen, Germany) with a peak emission at 313 nm was used for the UVB irradiation. The UVB output was measured using a Waldmann UV Meter (HerbertWaldmann GmbH & Co.). The UVB irradiation dose was controlled by adjusting the exposure time. Prior to irradiation, the cells were rinsed twice with pre-warmed PBS after removal of the media. The cell exposure to the UVB irradiation was performed in petri dishes containing PBS without sodium bicarbonate, which was immediately replaced by fresh DMEM containing 10% FBS after the UVB irradiation. The UVB irradiation dosages used in this study were 0, 25, 50, 75, 100, and 125 mJ/cm^2^. The temperature was kept constant during the irradiation procedure. The controls followed the same schedule of media changes without the UVB irradiation.

### RNA extraction

Total RNA was extracted from the fibroblasts 24 h after exposure to the different doses of UVB using TRIzol Reagent (Invitrogen, USA). In addition, total RNA was extracted from fibroblasts 12, 24, 36, and 48 h after exposure to a UVB dose of 125 mJ/cm^2^. Genomic DNA contamination was removed by on-column digestion using the RNAse-free DNase set (Qiagen, Germany). A Nanodrop ND1000 spectrophotometer (Gene, American) was employed to analyse the RNA concentration and purity. The absence of inhibitors was tested using 10-fold serial dilutions of pooled target gene cDNA. The integrity of the isolated total RNA was assessed with the RNA 6000 Nano LabChip^® ^kit using the Agilent 2100 Bioanalyzer (Agilent Technologies, USA). The Agilent 2100 Expert software was used to generate a so-called RNA Integrity Number (RIN) as an indicator of the RNA quality for downstream experiments. All samples were stored at -80°C and utilised within 2 months.

### cDNA synthesis

Purified and frozen RNA (800 ng) was reverse-transcribed in a 20 μl mixture using the RevertAid™ First Strand cDNA Synthesis Kit Oligo dT Primers (Fermentas, Canada) on a BioRad PTC-200 DNA Engine thermal cycler (BioRad, Hercules, USA). Briefly, the RNA samples and oligo(dT) primers were mixed and denatured at 70°C for 10 min. The tubes were then immediately placed on ice for at least 1 min. The transcription mixture and RNase inhibitor were added, and the mixture was incubated at 37°C for 5 min. The first-strand cDNA synthesis was started after the addition of M-mulv, and the reverse transcriptase reaction was performed at 42°C for 1 h. Finally, the enzymes were inactivated at 70°C for 10 min. The reactions were performed in triplicate to reduce any differences in the efficiency of the reverse transcription reaction. The cDNA was stored at -80°C and diluted 1:5 with RNase-free water for use as the template in the real-time PCR analysis.

### Primer design

The primers were designed using Primer 3 software (http://frodo.wi.mit.edu/primer3/) with the following parameters: melting temperature between 57°C and 63°C, length of 20 or 21 bp, GC content between 45% and 60%, and production of PCR products between 127 and 205 bp. The primers were tested for specificity using NCBI BLAST (http://www.ncbi.nlm.nih.gov/tools/primer-blast/), and Primer 5 was subsequently used to evaluate the target sequences amplified by the primer pairs to avoid the formation of secondary structures at the primer binding site. Primer specificity was confirmed by 2% agarose gel electrophoresis (results not shown) and by the melting curve analysis. The primer sequences and expected amplicon sizes are given in Table 2.

### Real-time PCR

The expression study was performed using a 96-well plate on an iQ5 iCycler Multicolor Real-Time PCR detection system (BioRad, Hercules, CA, USA) with the TransStart Green qPCR SuperMix UDG kit (TransGen Biotech, China). The reaction was performed according to the manufacturer's instructions with minor modifications. Briefly, the samples were amplified in 25 μl aliquots containing cDNA (2 μl), 1×IQ SYBR Green Supermix and 100 pmol each of the forward and reverse primers. The cycling conditions were 2 min at 50°C for the UDG incubation and 10 min at 95°C for the polymerase activation, followed by 40 cycles of denaturation at 95°C for 15 s, annealing at 55°C (the annealing temperatures for the different primers are given in Table 2) for 30 s and extension at 72°C for 30 s. The specificity analysis of the PCR products (melting curve analysis) was performed immediately after the real-time PCR. The temperature range used for the melting curve generation was 60°C to 95°C. Each sample was analysed in triplicate wells and was always analysed in one analytical run to avoid between-run variations. Each PCR run included a no-template control using water instead of cDNA.

A 10-fold dilution series was created from a cDNA pool from our sample group (including all samples) ranging from a 10× dilution to a 100000× dilution. Triplicate RT-qPCR reactions were carried out for each gene at each dilution. The mean cycle threshold (Ct) values for each dilution were plotted against the log_10 _of the cDNA input for each gene to generate the efficiency plots. The reaction efficiency for each gene assay was calculated using the following equation: E = 10^(-1/slope)^, where E is the reaction efficiency and 'slope' is the slope of the line generated in the efficiency plot.

### Statistical analyses

The average Ct value from the triplicate PCR performed for each sample and the average efficiency calculated from all samples for each gene were used. The mean Ct values of the replicates for each sample were transformed into raw, non-normalised quantities (Q) using the comparative ΔCt method by the equation Q = E^ΔCt^, where E is the reaction efficiency for each gene assay in question and ΔCt = min Ct - sample Ct, where min Ct is the lowest Ct value over a range of samples for a given assay, and sample Ct is the Ct value of the sample being transformed.

All statistical evaluations were carried out using the SPSS (version 17) software package. One-way ANOVA was used to test the gene expression differences between normal and irradiated fibroblasts. Significant results from the ANOVA tests were further analysed by Tukey's post-hoc test. P < 0.05 was considered significant.

The expression data was analysed using the geNorm algorithm, which determines a reference gene stability factor (M). The stability factor M was defined as the average variation of a particular gene compared with all of the other candidate reference genes. In the final analysis, genes with an M value lower than 0.5 were considered to be stably expressed genes. The candidate reference gene expression stability was also analysed by the NormFinder programme.

## Authors' contributions

LL was the main contributor to the manuscript and performed the majority of the experiments, data analysis and interpretation, and study design. LL also wrote the majority of the manuscript. YY and HXX collected the samples, participated in the study design and performed some of the experiments. TQ made critical contributions to the data analysis. BXW provided guidance in the study design and assisted with the data analysis. All authors were involved in critically revising the manuscript during its production, and they all read and approved the final manuscript.
